# Medication Risk Management in Routine Dispensing in Community Pharmacies

**DOI:** 10.3390/ijerph17218186

**Published:** 2020-11-05

**Authors:** Sonja Kallio, Tiina Eskola, Marika Pohjanoksa-Mäntylä, Marja Airaksinen

**Affiliations:** 1 Division of Pharmacology and Pharmacotherapy, Faculty of Pharmacy, University of Helsinki, 00014 Helsinki, Finland; tiinahakala2@gmail.com (T.E.); marika.pohjanoksa@helsinki.fi (M.P.-M.); marjaairaksinen@gmail.com (M.A.); 2 Hyvinkää 3rd Pharmacy, 05830 Hyvinkää, Finland; 3 Forssa 1st Pharmacy, 30100 Forssa, Finland

**Keywords:** medication management, medication risk management, community pharmacy, optimizing prescribing, potentially inappropriate prescribing, adherence, polypharmacy, medication safety

## Abstract

Community pharmacists have a duty to contribute to medication risk management in outpatient care. This study aimed to investigate the actions taken by pharmacists in routine dispensing to manage medication risks. The study was conducted as a national cross-sectional online survey targeted at all community pharmacies in Finland (*n* = 576) in October 2015. One pharmacist from each pharmacy was recommended to be the spokesperson for the outlet to describe their practices. Responses were received from 169 pharmacies (response rate of 29%). Pharmacists were oriented to solving poor adherence and technical problems in prescriptions, whereas responsibility for therapeutic risks was transferred to the patient to resolve them with the physician. Pharmacists have access to a wide range of electronic medication risk management tools, but they are rarely utilized in daily dispensing. Attention was paid to drug–drug interactions and the frequency of dispensing with regard to high-risk medicines. Pharmacies rarely had local agreements with other healthcare providers to solve medication-related risks. In routine dispensing, more attention needs to be given to the identification and solving of therapeutic risks in medications, especially those of older adults. Better participation of community pharmacists in medication risk management requires stronger integration and an explicit mandate to solve the therapeutic risks.

## 1. Introduction

Medication risk management is a strategy that aims to prevent or decrease risks associated with the use of medicines [1]. A foundation for safe medication use is laid by ensuring safe and effective pharmaceutical products are available and are appropriately prescribed and dispensed to patients, along with their effects being properly monitored [2]. National legislation and guidelines set the foundation for routine actions and procedures to be followed when prescribing and dispensing medicines in outpatient care. These safety actions need to consider that medications are primarily managed at home by patients themselves or with the assistance of their proxies. Special actions are needed to prevent the harm that medicines can cause to more vulnerable patient groups such as children, older adults, and people with multiple diseases and using multiple medications [3].

Furthermore, some medicines can pose a higher risk of harm to their users, and, thus, need special actions as part of their routine prescribing, dispensing, and monitoring (e.g., anticoagulation therapies, insulin, and psychotropic medications) [2,4,5]. Several classification systems for medication-related risks have been created for use in practice and research [6,7,8]. Poor medication risk management may lead to preventable adverse events that can impair health and quality of life as well as increase mortality, morbidity, hospitalization, and the economic burden to society [3,9,10].

All healthcare professionals involved in the medication-use process are responsible for medication risk management [11]. Community pharmacists have a special responsibility for dispensing medicines safely to outpatients and supporting their safe use at home and other outpatient settings. Routine dispensing has been extended to include the double-checking of doses and indications, identifying potentially harmful interactions and other medication-related risks that may pose preventable harm. Even though community pharmacists contribute to medication risk management in many ways, little is known regarding what risk management actions they actually take as part of routine dispensing [12,13,14]. Our recent systematic review indicated that medication review interventions involving community pharmacists could reduce medication-related risks and increase adherence in older adults [15]. The same systematic review also concluded that community pharmacists could contribute more to patient care than is currently the case, and more evidence should be available on the impact of the interventions of community pharmacists on medication risk management. This study aimed to investigate the contributions of community pharmacists to medication risk management in Finland and to clarify what risk management actions are actually taken as part of routine dispensing.

## 2. Materials and Methods

In Finland, community pharmacies are the only source of prescription and non-prescription medicines in outpatient care [16]. Medicines are primarily prescribed and renewed by physicians. Medications for most of the long-term therapies can be prescribed for a 2-year supply without scheduled follow-ups in the middle. The patients with chronic conditions visit their community pharmacy at least every third month to pick up their medicines for the next three months’ supply allowed to be dispensed at a time by the public reimbursement scheme covering the entire population [17].

The supply of medicines to outpatients is provided by approximately 600 privately owned community pharmacies and by two teaching pharmacies run by the University of Helsinki and University of Eastern Finland [16]. All medicines need to be dispensed in their original packages, labelled, and equipped with package leaflets as required by the European Union regulation [18]. When dispensing, pharmacists are obliged to ensure that medicine users are aware of how to use their medicines safely and appropriately. Most of the community pharmacies have access to the same health and medicine information databases and medication risk management tools as physicians and other healthcare providers [19,20,21]. However, pharmacists are not allowed to make any changes to prescribed medications without consulting the prescribing physician [16]. Community pharmacies increasingly offer services that support the rational use of medicines, automated dose dispensing being the most common service available [22]. The tendency toward more enhanced integration of community pharmacies into the healthcare system has been continuously supported by medicine policy initiatives [23,24].

This national cross-sectional online survey was targeted at all community pharmacies in Finland. The invitation to participate in the study was sent by email to the member pharmacies of the Association of Finnish Pharmacies (*n* = 574) and university pharmacies (*n* = 2) in October 2015. One pharmacist from each pharmacy was recommended to be the spokesperson on behalf of the outlet. Respondents were given two weeks to make contact. A reminder email was sent a week after the invitation to participate in the study. As the response rate was still low (24%) two weeks after sending the survey, another reminder email was sent in which the response time was extended by one week.

Reason’s theory of human error was applied as a theoretical framework for the study [25]. According to the theory, risk management actions taken in routine dispensing can be considered as defenses to prevent potential medication-related risks and errors from occurring.

An online survey instrument was developed to assess the community pharmacists’ risk management actions taken in routine dispensing practice. The final instrument was primarily structured with some open-ended, probing questions. This survey consisted of the following three themes: (1) actions taken in routine dispensing in the community pharmacy to manage medication-related risks; (2) actions taken to manage risks related to use of high-risk medications; and (3) how systematically these risk management actions are taken in routine dispensing.

The present study applied four of the survey questions. Actions taken in routine dispensing to manage medication-related risks were assessed with the following two questions: (1) How does your pharmacy primarily address the following medication-related risks in routine dispensing? and (2) Does your pharmacy have in-house agreements or agreements with your local healthcare providers for the following situations? Both of the questions had a structured list of options for response. Medication-related risks listed in the survey were adapted from the classification of the Pharmaceutical Care Network Europe (The PCNE Classification V 6.2) [26]. In the first question, the response options were as follows: (1) by discussing with the patient; (2) by discussing with the patient and advising them to contact the physician when needed; (3) by advising the patient to contact the physician; (4) by contacting the physician; (5) by offering prescription review or medication review for the patient; (6) no action; and (7) by some other means. The options for responses in the second question were: (1) the pharmacy has an in-house agreement; (2) the pharmacy has a joint agreement with local healthcare providers; (3) the pharmacy has both an in-house agreement and a joint agreement with local healthcare providers; and (4) no agreed-upon approaches.

The second theme concerning the management of high-risk medications had the following question: In addition to normal dispensing routines, do you pay special attention to the following high-risk medicines? Selected high-risk medicines were based on the classification of high-alert medications in community/ambulatory care by the Institute for Safe Medication Practices [27]. The following options to respond were given: (1) special attention is paid, and (2) no special attention is paid. Respondents were invited to elaborate in open fields what actions they took to manage the risks related to each of the high-risk medicines.

The third theme concerned risk management actions to identify medication-related risks and applied the following structured question: How is a potential medication-related risk typically identified in your pharmacy? Options to respond were: (1) with the help of the electronic drug-drug interaction database; (2) by checking the prescription (e.g., double-checking the dose); and (3) by discussing with the patient about their health and the success of the medication self-management. In each of these options, the respondents were required to state how systematically the action was taken in their pharmacy. The options were: (1) systematically with all patients; (2) selectively with some special patient groups (e.g., older adults) or patients with high-risk medications (e.g., warfarin); (3) each pharmacist can act in their own way; and (4) action is not taken in our pharmacy.

As we did not find any previously used validated survey instruments focusing on our research question from the literature, we developed a new one. We applied general principles of scale development and validation, starting from item development, coming up with the initial set of questions for an eventual scale, and evaluating the eventual scale for content and face validity [28]. The item generation was based on (1) principles of systems-based medication safety with the emphasis on prospective medication risk management in community pharmacy context, and (2) regulations and recommended best practices guiding routine dispensing in this respect. The face and content validity of the survey instrument was assessed by two practicing pharmacists from two community pharmacies and an expert pharmacist from the Association of Finnish Pharmacies [28]. After including their comments, the survey instrument was piloted by four community pharmacists. Minor clarifications and modifications were made in the questions using their comments.

Descriptive quantitative analysis was conducted using IBM SPSS Statistics version 22.0 [29]. Responses to the open-ended questions concerning high-risk medicines were analyzed qualitatively using content analysis [30].

The research was conducted according to good ethical and scientific practice as set by the Finnish National Advisory Board on Research Integrity [31]. Ethics committee approval was not required for this kind of health service research, which did not include medical interventions to patients [32]. The anonymity of the participating pharmacists was ensured, and they were informed that their responses would be used only for research purposes. The study participation was voluntary, and responding to the survey was considered to be as giving consent to participate.

## 3. Results

Responses were received from 171 community pharmacies, of which 169 responses were included in the study (response rate of 29%). Two of the responses were excluded because of an unidentified technical error in the documentation of their responses in the electronic survey system. A majority (80% in total) of the respondents were pharmacy owners (43%) or MSc pharmacists who usually performed as managers (37%) (Table 1). Most of the respondents (78%) worked in small- or medium-sized pharmacies dispensing less than 100,000 prescriptions annually.

### 3.1. Medication Risk Management Actions Taken in Routine Dispensing

**Figure 1:**
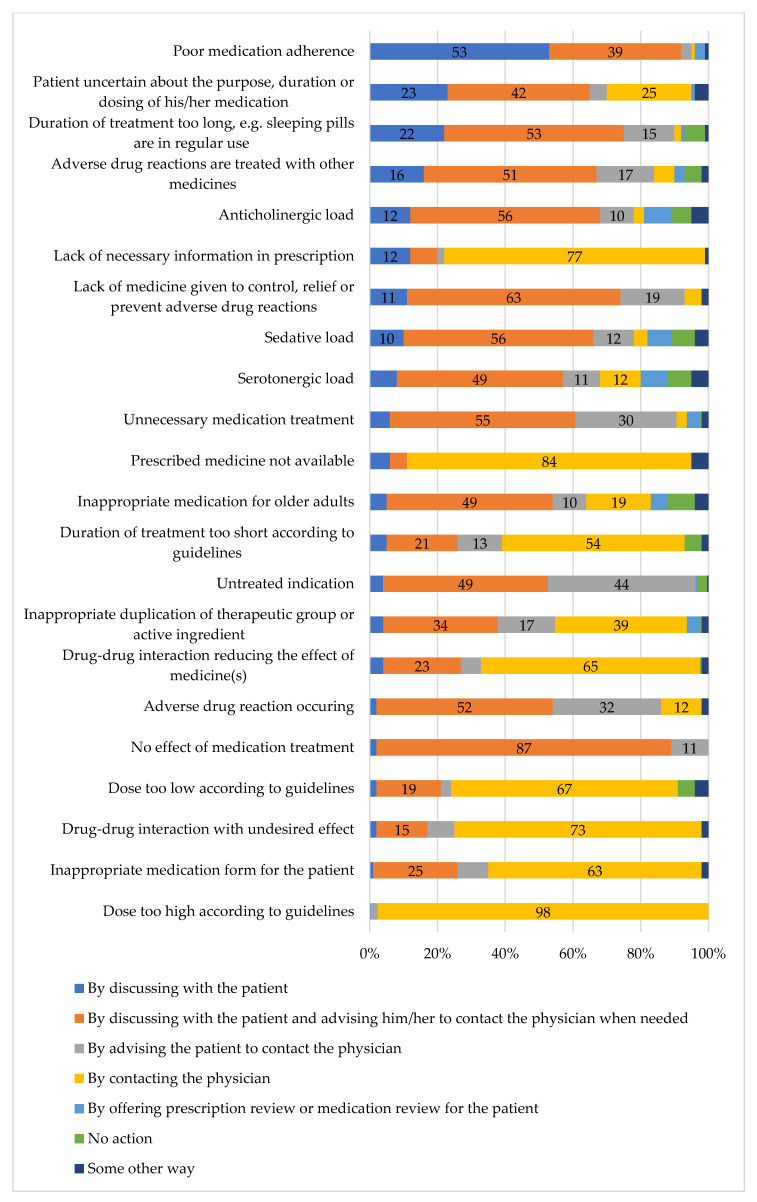
Strategies to solve medication-related risks during daily dispensing in community pharmacies (% of the responding pharmacies, *n* = 169. Responses that were given by less than ten per cent of respondents have not been included).

The community pharmacists who responded on behalf of their outlets (*n* = 169) reported three common approaches to act on medication-related risks (Figure 1). The most common action was to discuss the risk with the patient and advise them to contact the physician for further review of the medication. This approach was most likely to be taken when the medication treatment was not effective (87% of the responding pharmacies) and when the medication caused anticholinergic load (56%) or sedative load (56%). The physician was mainly consulted directly from the pharmacy when some technical aspect was wrong with the prescription, for example, the dose deviated from the recommended dose in the current guidelines (98% of the respondents reported contacting the physician if the dose was too high, 67% if the dose was too low) or necessary information was missing from the prescription (77%). In more than half of the pharmacies (53%), poor medication adherence was reported to be managed by discussing it with the patient without consulting the physician.

### 3.2. In-House and Local Agreements for Solving Medication-Related Risks

**Figure 2:**
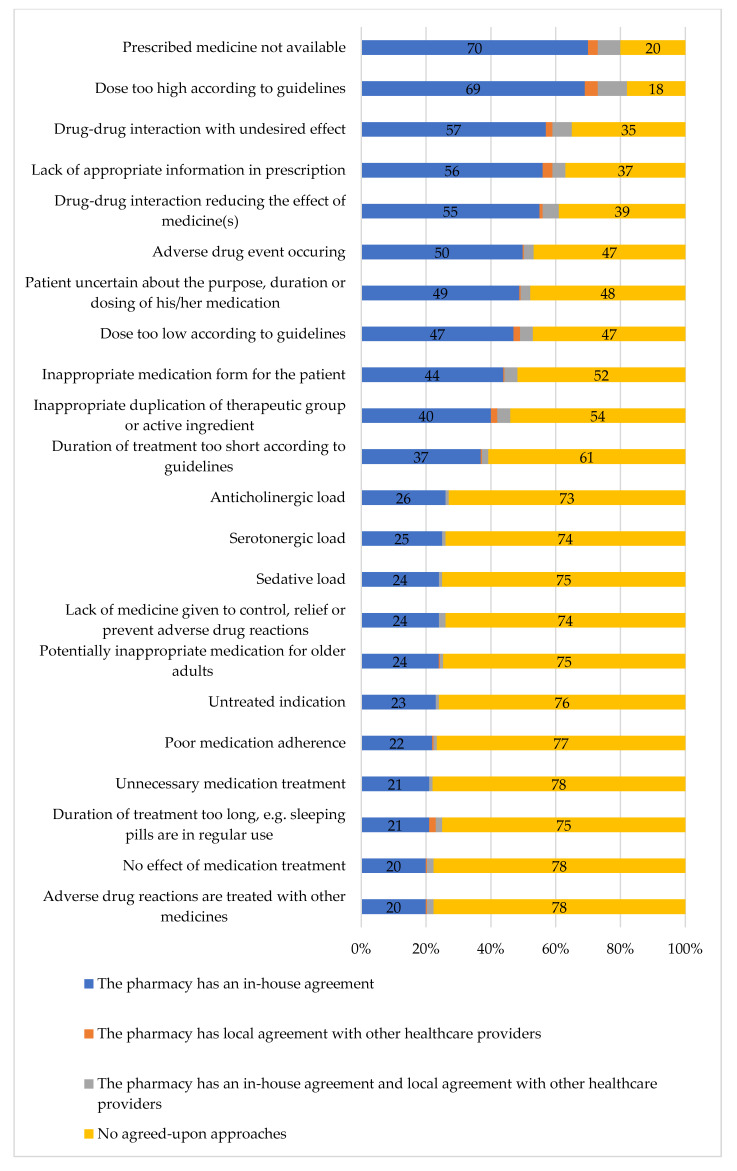
In-house and local agreements for managing medication-related risks in community pharmacies (% of the responding pharmacies, *n* = 169. The responses that were given by less than 10 per cent of respondents have not been included).

The approaches to managing different medication-related risks varied (Figure 2). Most commonly, pharmacies had in-house agreements for situations where the medicine prescribed was not available (70% of the responding pharmacies), and for solving risks caused by technical deficiencies in the prescriptions such as too high a dose compared with the recommended standard dose (69%) or too low a dose (47%), and when the prescription missed some information required to be filled and dispensed (56%). Of the therapeutic risks, pharmacies most commonly had in-house agreements for managing risks caused by clinically significant drug–drug interactions (57% of the responding pharmacies). For other therapeutic risks, pharmacies seldom had in-house agreements.

The majority of the pharmacies did not have agreements with local healthcare providers for managing and solving medication-related risks (Figure 2). The range of agreements varied between 1–13%. Moreover, a high number of pharmacies reported not having any in-house or local agreements in this respect. The number of pharmacies without agreements increased toward managing therapeutic risks and problems in the medications such as anticholinergic, serotonergic, or sedative loads (73–75% of the pharmacies reported no agreements) or inappropriate medications for older adults (75%).

### 3.3. Management of High-Risk Medications in Routine Dispensing

**Figure 3:**
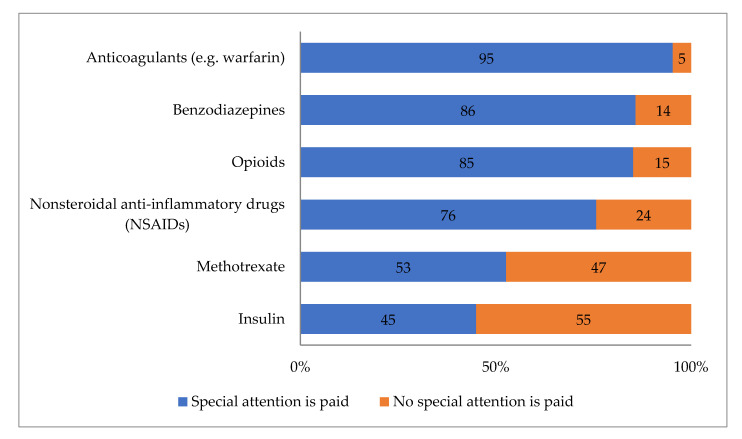
Management of high-risk medications in routine dispensing (% of the responding pharmacies, *n* = 169).

Of the high-risk medicines included in the survey, the respondents most commonly reported paying special attention to anticoagulants (95% of the pharmacies), benzodiazepines (86%), and opioids (85%) (Figure 3). In open fields, the respondents reported that, for anticoagulants, special attention was most commonly paid to interactions between the anticoagulant and other medicines or food (70% of all pharmacies). For benzodiazepines and opioids, they reported following the frequency of dispensing most commonly (39% benzodiazepines and 30% for opioids). Drug–drug interactions were reviewed when dispensing nonsteroidal anti-inflammatory drugs (NSAIDs) (40%). One-third (33%) reported that they discussed potential adverse drug reactions of NSAIDs, most frequently gastrointestinal symptoms, with the patients while dispensing. The most commonly discussed issue with the methotrexate users was reported to be the correct dosage interval (31%), while, for insulin, it was doses (16%).

### 3.4. Actions Taken to Identify Medication-Related Risks in Routine Dispensing

**Figure 4:**
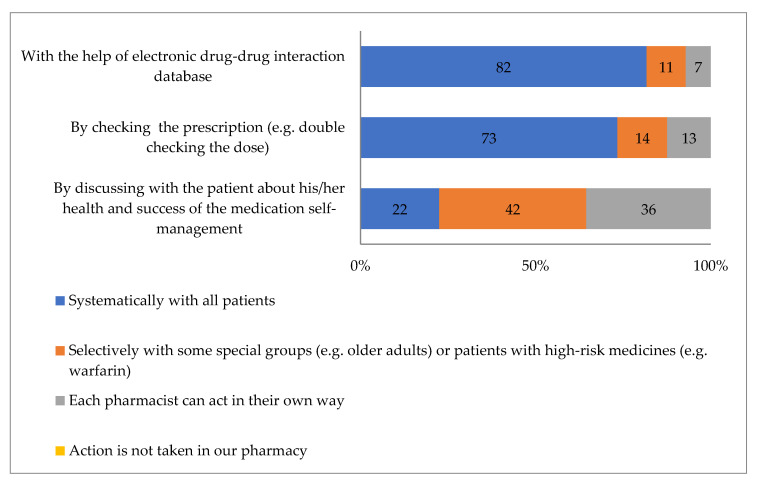
Actions taken to identify medication-related risks in routine dispensing in community pharmacies (*n* = 169).

In the majority of the pharmacies (82%), all prescriptions were reported to be reviewed routinely as part of the dispensing with the help of the electronic drug–drug interaction database (Figure 4). Individual prescriptions were reviewed for proper dosing in 73% of the pharmacies. Discussion with the patient about their health and the success of the medication self-management was not routinely performed. However, it was merely targeted to selected clients such as older adults or clients using high-risk medications. The practices of discussion with the patients also differed between pharmacists.

## 4. Discussion

Our findings indicate that Finnish community pharmacists contribute to medication risk management to some extent, but that their contributions could be increased. The routine risk management actions of pharmacists seem to be concentrated on reviewing the prescriptions from a technical and legal perspective to ensure their compliance with prescribing, dispensing, and reimbursement rules. These rules oblige pharmacists to double-check the dose regimen and indication, control overuse, particularly of psychotropic and chronic medications, and ensure that the medicine user is aware of how to use the medicine [33]. Pharmacists are independently allowed to correct only technical errors in prescriptions, but not to make any therapeutic decisions and changes without consulting the physician, except for generic substitution [33]. It seems that although some potential medication-related risks can be identified in routine dispensing, they are rarely solved by community pharmacists. Therapeutic risks in medications are principally discussed with the patients, and the pharmacists recommend that patients contact their physicians to solve therapeutically significant risks (e.g., when the medication is not effective or causes severe adverse reactions or if potentially inappropriate medications for older adults are in use).

Pharmacists seem to have acquired a more independent role in managing risks related to poor adherence through discussions with the patients. This dialogue between pharmacists and patients is often based on regular encounters that could be utilized even more strongly in follow-ups of medication treatments. Our study did not assess the pharmacists’ actions to manage risks related to inappropriate self-management of treatments at home. This important area of future research as risks related to self-management are not well-understood, even though they have a crucial impact on the outcomes of pharmacotherapies [34,35,36].

Drug–drug interactions seemed to be therapeutic risks most routinely and systematically screened in community pharmacies while dispensing. This fact may be related to the fact that the drug–drug interaction screening tool was the first electronic screening tool for medication risk management that was launched in Finnish healthcare including community pharmacies in 2004 [19]. Since then, the other risk management tools integrated into computerized physician order entries and prescription processing systems in community pharmacies have been made widely available [21,37]. These tools cover, for example, drug-induced adverse reactions, anticholinergic and serotonergic load, potentially inappropriate medicines for older adults, and medication safety during renal or hepatic failure, pregnancy, or lactation. However, their routine use seemed to be in its infancy in community pharmacies when the data for this study were collected in 2015. Further research should be focused on understanding the factors related to the limited use of these advanced medication risk management tools in routine dispensing.

When reflecting upon the results of our study in the context of major public health concerns in Finland, community pharmacists should primarily enhance their contributions to managing medication risks, most prominently in older adults with multiple diseases and multiple medications. Such risks include excessive polypharmacy, potentially inappropriate medication use, high-risk medication use, and burden of severe adverse drug reactions (e.g., serotonergic and anticholinergic loads) [38,39]. In addition to not using the risk management tools available effectively, more active involvement of community pharmacists may be hindered by the lack of joint agreements within the pharmacy and between the pharmacy and local healthcare providers on each one’s duties and responsibilities.

The lack of agreements may be due to the physical separation of community pharmacies from other healthcare units. It may also reflect traditional hierarchical thinking and perception of the pharmacy as a logistical distribution point for pharmaceuticals, and not as a healthcare unit [40,41,42]. This point may still be the case even though community pharmacies have remarkably increased their capacity as part of the health service system in terms of equipment, facilities, and competence of the personnel. According to a recent national study, community pharmacy owners are willing to invest in providing health-oriented services [22]. This tendency has been continuously supported by the national medicines policy [22,24]. However, the realization of these policies has been challenging as the financial structure of pharmacies in Finland is still primarily based on the sales of medicinal products. Pharmacies are not paid for providing cognitive services, as has been done, for example, in the UK where accredited pharmacy services such as Medicines Use Review (MUR) and new medicine service are included in the National Health Service (NHS) scheme [43,44], along with Medication Therapy Management (MTM) in the U.S. [45].

This study is the first nationwide study assessing medication risk management in routine dispensing in community pharmacies. Previous studies on community pharmacists’ involvement in medication risk management have focused on managing drug–drug interactions, particularly implementing screening and alert systems for their identification [13,14,19]. This study extended the scope to a wider range of medication-related risks common in outpatient care such as managing risk load and high-risk medications and patients [4,10,46]. The scope of the study was prospective risk management, also covering the prevention of risks related to medication self-management. Furthermore, the study was extended to assess what actions community pharmacists took to prevent and solve medication-related risks at the individual patient level, how systematically they took their risk management actions, and how they collaborated with other healthcare providers and patients in this respect.

This national cross-sectional study had a low response rate, which decreases the power of the study and generalization of the findings. It is likely that the responding community pharmacies were more therapeutically oriented and had medication risk management as a priority in their operations compared to the non-respondents. Thus, the results may give too positive an overall image of the Finnish community pharmacists’ involvement in medication risk management. The survey instrument proved to be valid and feasible to use. Its further use is recommended, for example, in a follow-up study to assess the evolution of medication risk management practices in community pharmacies. The instrument is currently utilized for self-assessing medication risk management skills of pharmacy students at the University of Helsinki. Having one informant from each pharmacy proved to be a feasible strategy for collecting the data on their practices. The spokesperson was most commonly the pharmacy owner, who had an MSc (Pharm) degree. The owners, as informants, were able to provide an overall picture of the operations of their pharmacies. Reports from staff pharmacists working in actual dispensing and customer service would have given another approach to medication risk management in Finnish community pharmacies.

This kind of national survey works as an indicative study of the state of the medication risk management practices in community pharmacies. The results can inform policymaking and practice development. They are useful for authorities, professional organizations, universities, continuing education providers, individual pharmacy owners and practitioners. The results also provide important information to those involved in planning the integration of pharmacy services in other health services as part of the ongoing social and health services reform [22,24]. As the data were collected in 2015, it is recommended to repeat the survey to follow up on the most recent developments. There is a growing need for community pharmacists’ involvement in medication risk management as populations are aging, particularly in Finland. The aging rate is one of the fastest in the world. This need for pharmacists’ involvement in the prospective prevention of medication-related harm has been recognized in recent national and international policies.

## 5. Conclusions

The solving of medication-related risks in community pharmacies is still minor and principally concentrates on technical issues related to prescriptions. More attention needs to be paid to the identification and solving of potential therapeutic risks in medications, especially those of older adults. Issues related to patients’ poor medication adherence can be managed by community pharmacists, while therapeutic risks are often left to be resolved by the patient with the physician. Better participation of community pharmacists in medication risk management requires an explicit mandate and stronger integration to solve therapeutic risks.

## Figures and Tables

**Table 1:** Characteristics of the respondents and their community pharmacies (*n* = 169). The percentages in brackets describe the situation of all pharmacies in Finland (*n* = 574). Data source: The Association of Finnish Pharmacies.

	*n*	%
**Work title and degree**		
Pharmacy owner, MSc (Pharm)	72	43 (12)
Pharmacist, manager, MSc (Pharm)	63	37 (15)
Dispensing pharmacist, BSc (Pharm)	34	20 (73)
**Annual prescription volume in 2014**		
<60,000 (small-sized pharmacies)	74	44 (43)
60,000–100,000 (medium-sized pharmacies)	57	34 (32)
>100,000 (large-sized pharmacies)	38	22 (25)
**Location**		
Southern Finland	51	30 (n/a)
Western Finland	70	41 (n/a)
Eastern Finland	21	13 (n/a)
Northern Finland	27	16 (n/a)
